# Coding, or non-coding, that is the question

**DOI:** 10.1038/s41422-024-00975-8

**Published:** 2024-07-25

**Authors:** Laura Poliseno, Martina Lanza, Pier Paolo Pandolfi

**Affiliations:** 1 Oncogenomics Unit, Core Research Laboratory, ISPRO, Pisa, Italy; 2 Institute of Clinical Physiology, CNR, Pisa, Italy; 3 University of Siena, Siena, Italy; 4 Department of Molecular Biotechnology and Health Sciences, Molecular Biotechnology Center, University of Turin, Torino, Italy; 5 Renown Institute for Cancer, Nevada System of Higher Education, Reno, NV USA

**Keywords:** Cancer models, Targeted therapies, Mechanisms of disease, Transcription, Translation

## Abstract

The advent of high-throughput sequencing uncovered that our genome is pervasively transcribed into RNAs that are seemingly not translated into proteins. It was also found that non-coding RNA transcripts outnumber canonical protein-coding genes. This mindboggling discovery prompted a surge in non-coding RNA research that started unraveling the functional relevance of these new genetic units, shaking the classic definition of “gene”. While the non-coding RNA revolution was still taking place, polysome/ribosome profiling and mass spectrometry analyses revealed that peptides can be translated from non-canonical open reading frames. Therefore, it is becoming evident that the coding vs non-coding dichotomy is way blurrier than anticipated. In this review, we focus on several examples in which the binary classification of coding vs non-coding genes is outdated, since the same bifunctional gene expresses both coding and non-coding products. We discuss the implications of this intricate usage of transcripts in terms of molecular mechanisms of gene expression and biological outputs, which are often concordant, but can also surprisingly be discordant. Finally, we discuss the methodological caveats that are associated with the study of bifunctional genes, and we highlight the opportunities and challenges of therapeutic exploitation of this intricacy towards the development of anticancer therapies.

## Introduction

In his article published in *Nature* in 1970, Francis Crick stated the central dogma of molecular biology: information is passed from DNA (gene) to protein, through messenger RNA (mRNA).^1^ Approximately 50 years later, the central dogma still stands. However, it has become just one among many mechanisms through which functional molecules are expressed from our genome.^2^

Over the years it has become more and more evident that our genomic DNA is pervasively transcribed. The portion transcribed into mRNAs, which in turn are translated into ∼20,000 proteins, is minimal (2%–5%). Far from being “junk DNA”, most of the rest (75%–90%) is rather transcribed into hundreds of thousands of non-coding RNAs (ncRNAs)^3^ that are emerging as sophisticated regulators of gene expression. Together with proteins, they govern embryonic development,^4,5^ maintain the physiological state,^6^ define organism complexity,^3,7^ and are causally linked with hereditary and non-hereditary diseases,^8,9^ including cancer.^5,10^

According to their function, ncRNAs are classified into housekeeping ncRNAs and regulatory ncRNAs. Housekeeping ncRNAs (ribosomal RNAs (rRNAs), transfer RNAs (tRNAs), small nuclear RNAs (snRNAs), and small nucleolar RNAs (snoRNAs)) were the first ncRNAs to be identified and studied. They are up to ~4.5 kb long and ubiquitously expressed, as they are involved in essential processes for cell survival. Specifically, rRNAs and tRNAs reside in the cytoplasm and are core components of the mechanism of protein translation. rRNAs represent the RNA component of ribosomes, while tRNAs act as adaptors between the mRNA and amino acids. snRNAs and snoRNAs are instead located in the nucleus. snRNAs represent the RNA component of spliceosomes. snoRNAs represent the RNA component of small nucleolar ribonucleoprotein particles (snoRNPs), which are responsible for maturation of pre-rRNAs through nucleoside modifications (mainly methylation and pseudouridylation).^11,12^ Interestingly, examples are accumulating of rRNAs, tRNAs, snRNAs, and snoRNAs with an oncogenic or tumor-suppressive role in human cancer, precisely because they regulate core mechanisms of gene expression. They are also explored as diagnostic and prognostic biomarkers.^13–15^

Regulatory ncRNAs are further divided according to their lengths. Among short ncRNAs (< 200 nt) there are PIWI-interacting RNAs^16^ and microRNAs (miRNAs). Long ncRNAs (lncRNAs, ≥ 500 nt) include long intergenic non-coding RNAs (lincRNAs), pseudogenic RNAs (PGs), Natural Antisense Transcripts (NATs), and circular RNAs (circRNAs).^5^ The expression of regulatory ncRNAs is tightly controlled, they perform a range of functions that is as wide as that performed by proteins, and their dysregulation has been linked to many pathological conditions, including cancer.

miRNAs are 19–25-nt short ncRNAs that inhibit gene expression at post-transcriptional level. The human genome contains hundreds of miRNAs (https://mirbase.org/). Primary miRNAs (pri-miRNAs) are transcribed and processed (first in the nucleus, then in the cytoplasm) to precursor miRNAs (pre-miRNAs) and finally to single-strand mature miRNAs, which recruit the Argonaute (AGO) protein to form the miRNA-induced silencing complex (miRISC).^10^ miRISC binds target RNAs in correspondence of specific sequences termed miRNA Recognition Elements (MREs). The result is a decrease in target expression through RNA degradation or translational repression. Each miRNA recognizes hundreds of targets, and each target can be recognized by multiple miRNAs. Therefore, most miRNAs are soft and pervasive tuners of gene expression.^17–19^ In addition, as described in “Translation of pri-miRNAs” chapter below, examples exist of miRNA precursors that are translated into functional peptides.

In cancer, miRNA expression and sequence are altered,^20^ and, depending on the repressed targets, they act as oncogenes (oncomiRs) or as tumor suppressors.^21–23^ Interestingly, there are cases in which the same miRNA behaves as oncogene in some cancer types and tumor suppressor in other cancer types. This attests the pervasive nature of miRNA-mediated regulation of gene expression, with net results depending on the balance among all targets at play.^24^ miRNAs are also extensively used as diagnostic, prognostic, and predictive biomarkers. Since they can be detected in body fluids such as blood, saliva and urine, circulating miRNAs represent in fact a much less invasive alternative to the classic biopsy.^25–27^ Finally, they are exploited therapeutically (inhibition of oncomiRs and replacement of tumor-suppressive miRNAs).^28^

Relevant to the topic of this review, we discovered a new mechanism for the regulation of miRNA activity: miRNAs are negative regulators of target RNA expression, but in turn target RNAs are negative regulators of miRNA function. This is because they compete for binding to common miRNAs, i.e., they act as competing endogenous RNAs (ceRNAs). ceRNA partners that share MRE(s) for the same miRNA(s) dilute the miRNA(s), blunting their efficacy and at the same time sustaining their own expression.^29^ ceRNA-based regulation can occur only if some conditions are satisfied: ceRNA partners and the shared miRNA(s) need to be present in the same subcellular compartment and to be expressed at similar level. Furthermore, the sponging effect gets stronger at the increase of the number and the affinity of shared MREs.^30–38^ Experimental evidence indicate that ceRNA-based regulation is common among coding (see “The 3′UTR exerts non-coding functions” chapter below) and all classes of non-coding RNAs. Furthermore, it can produce extended networks, which get heavily disrupted in cancer.^39–47^

The human genome contains more than 30,000 lncRNA genes, which are expressed into more than 100 thousand transcripts (https://www.gencodegenes.org/human/stats_45.html^5,48^). Although the mechanism of transcription initiation and termination can be different from those of mRNAs,^49^ most lncRNAs are transcribed by Pol II. Nevertheless, examples exist of lncRNAs transcribed by Pol I or Pol III. Furthermore, many lncRNAs undergo splicing, but they can be transcribed from single-exon genes as well.^50^ Analogously, they may or may not undergo 5′ capping and 3′ polyadenynation.^5^ Besides transcription mechanisms, heterogeneity extends to many other features of lncRNAs. According to the position of their genes, they are grouped into lincRNAs, intronic lncRNAs or pseudogenes, while according to the direction of their transcription they are divided into sense transcripts and NATs. Furthermore, all lncRNAs are linear transcripts, except for circRNAs, while subcellular localization defines nuclear vs cytoplasmatic lncRNAs.^5,51^

To date, just a small fraction of all lncRNAs have been studied, and what we already know might just be the tip of the iceberg. In any case, they appear as flexible molecules that organize in thermodynamically-stable secondary or even higher-order structures. Acting as signals, guides, decoys, or scaffolds, they regulate virtually each step of gene expression: nuclear organization and genome integrity, chromatin remodeling by epigenetic modification, transcription, RNA splicing and processing, mRNA stability and translation, protein post-translational modification (e.g., phosphorylation), subcellular localization, and activity.^5,52–55^

lncRNAs are altered in cancer and, depending on their mechanisms of action and on the effectors involved, they can act either as oncogenes or as tumor suppressors. Furthermore, in the last few years lncRNAs have taken center stage as diagnostic, prognostic and predictive biomarkers. Crucially, oncogenic lncRNAs are currently explored as therapeutic targets, using approaches based on antisense oligonucleotides, RNA interference, or CRISPR/Cas9 technology.^56,57^ Furthermore, lncRNAs encoding tumor-specific antigens can be exploited as anticancer vaccines.^58^

Among linear lncRNAs, lincRNAs are the most conspicuous group and include some of the most well-studied examples, in development and in cancer.^59^ LincRNA *X-inactive specific transcript* (*Xist*), which is responsible for X chromosome inactivation and gene dosage compensation, was discovered in the early 90s and is the first long non-coding RNA to be ever studied.^60^ Fifteen years later, *HOX antisense intergenic RNA* (*HOTAIR*) was discovered. This lincRNA is transcribed from an independent promoter located in antisense strand within the *HOXC* locus, and it causes epigenetic silencing of the *HOXD* locus.^61^ In cancer, examples of well-studied lincRNAs include: *BRAF-Activated Non-protein Coding RNA* (*BANCR*^62^), *linc-Regulator Of Reprogramming* (*linc-RoR*^63^), *Metastasis-Associated Lung Adenocarcinoma Transcript 1* (*MALAT-1*^50^), and *Survival Associated Mitochondrial Melanoma Specific Oncogenic Non-coding RNA* (*SAMMSON*^64^). These are pleiotropic lincRNAs that exert oncogenic functions in multiple cancer types by affecting key cellular processes (motility, stemness, chemosensitivity, and mitochondrial metabolism, respectively). Mechanistically, lincRNAs have the activities listed above for lncRNA. Interestingly, they can also be translated into functional peptides, as we describe in “Translation of lincRNAs” chapter below.

A group of linear lncRNAs with peculiar features is represented by NATs. The development of high-throughput sequencing has shed light on this class of RNA molecules that are transcribed from the opposite DNA strand. They originate from bidirectional promoters shared with the corresponding sense transcripts, from independent antisense promoters, or from latent antisense promoters located within sense transcriptional units. According to the degree of overlap with sense transcripts, NATs are defined as head-to-head (the overlap is in the 5′ region), tail-to-tail (the overlap is in the 3′ region) or embedded (the overlap is complete). Finally, *cis*-NATs exert their function on their own genomic locus, while *trans*-NATs act on other genomic loci. In cancer, several NATs are known to be involved in the regulation of gene expression at multiple levels: in the nucleus, epigenetic modification, transcription, RNA splicing and processing; in the cytoplasm, mRNA stability and translation, post-translational modification of proteins. As such, they play oncogenic or tumor-suppressive roles and they are highly valued as diagnostic, prognostic, and predictive biomarkers.^65,66^

Another group of linear lncRNAs with peculiar features is represented by pseudogenes. With the name “pseudogene” we refer to a region of the genome that contains a defective copy of a parental protein-coding gene. Indeed, pseudogenes are characterized by the presence of mutations, deletions or insertions that lead to frameshifts and prevent translation of parental protein products. The human genome contains ∼14,000 pseudogenes, 10% of which are transcribed (https://www.gencodegenes.org/human/stats_45.html^67^).

According to their origin, pseudogenes are classified as follows. Processed pseudogenes, which represent the most abundant class, derive from a retrotransposition event. They do not contain introns, are located on different chromosomes compared to parental genes, and are subjected to a distinct regulation of gene expression. They accumulate alterations because retrotranscription is error prone. Nonprocessed pseudogenes derive from gene duplication. They are located on the same chromosome as parental genes and retain introns, as well as regulatory regions (promoter). They accumulate alterations because the sequence of the parental copy is the only one preserved under selective pressure. Finally, unitary pseudogenes arise from the accumulation of alterations in an ancestral protein-coding gene that has no other copy in the genome.^68,69^

Even though they have been considered functionless for a long time, in the recent years we and others have contributed to discovering that pseudogenes exert a wide range of parental gene-related as well as parental gene-unrelated functions. Specifically, PGs are involved in chromatin remodeling, sponging of miRNAs and RNA Binding Proteins (RBPs), and mRNA degradation through endosiRNAs.^68,70–74^ As described in “Translation of PGs” chapter below, they are also translated into functional peptides and proteins.

The contribution of pseudogenes to cancer initiation and progression is gaining momentum. There are in fact several examples of oncogenic and tumor-suppressive pseudogenes.^69^ Interestingly, with their random “landing” upon retrotranscription, processed pseudogenes acquired somatically can potentially disrupt otherwise functional genetic units and therefore can be considered mutagenic factors.^75^ PGs are also highly valuable as diagnostic and prognostic biomarkers.^76^ Oncogenic PGs are explored as therapeutic targets,^77,78^ while PGs that act as sponges of oncogenic miRNAs are envisioned as drugs.^73^ Finally, PGs that encode immunogenic peptides could reveal effective as anticancer vaccines.^73,79^

The ∼25,000 circRNAs constitute the group of non-linear lncRNAs.^80^ circRNAs are single-stranded, covalently-closed RNA molecules characterized by high stability since they are immune from exonucleases activity. circRNAs are mostly generated by back-splicing of pre-mRNAs. Contrary to canonical splicing in which an upstream 5′ splice donor is joined with a downstream 3′ splice acceptor, back-splicing is an unconventional splicing event in which an upstream 3′ splice acceptor is joined with a 5′ downstream splice donor, leading to the formation of a circular-shaped structure. As a result of this peculiar splicing mechanism, circRNAs are classified as follows. Exonic circRNAs (EcircRNAs) are composed entirely by exons. They are the largest subclass of circRNAs (they account for ∼85% of all circRNAs) and are mainly located in the cytoplasm. Conversely, Exonic-Intronic circRNAs (EIcircRNAs) are composed both by exons and by introns and are mainly retained in the nucleus. In alternative to back-splicing, circRNAs can also originate from lariat introns that fail to undergo debranching and are subsequently subjected to trimming of the lariat tail. Because of such biogenesis, circular intronic RNAs (ciRNAs) are composed only by introns and are mainly retained in the nucleus.^81^

The molecular functions attributed to circRNAs are in line with their subcellular localization. Nuclear EIcircRNAs and ciRNAs mainly act *in cis* as regulators of transcription or splicing of their own gene. Conversely, cytoplasmic EcircRNAs mainly act *in trans*: as sponges for miRNAs and for RBPs, they play a crucial role in the post-transcriptional regulation of gene expression; they form circRNPs that modulate signaling pathways; as described in “Translation of circRNAs” chapter below, they can also be translated into functional peptides.^82,83^

There are several examples of oncogenic or tumor-suppressive circRNAs in cancer.^84^ They have gained attention in the context of resistance to traditional chemotherapeutic drugs, as well as targeted and immunotherapy approaches.^85,86^ Extensively studied as diagnostic, prognostic, and predictive biomarkers,^83,87^ circRNAs are the focus of many therapeutic strategies as well. Oncogenic circRNAs are targeted through antisense oligonucleotides, RNA interference, or CRISPR/Cas9 technology. In addition, their exceedingly high stability has prompted testing of circRNAs as drugs: synthetically engineered circRNAs are currently under evaluation as sponges for oncogenic miRNAs,^83,87,88^ while circRNAs encoding tumor-specific antigens can be envisioned as anticancer vaccines.^89,90^

Still overwhelmed by the discovery of pervasive genome transcription, in the most recent years we have been hit by a counter-wave of pervasive translation. In the last decades, the ∼20,000 proteins encoded by our genome (https://www.gencodegenes.org/human/stats_45.html) have been the undivided focus of cancer research. The most potent oncogenes and tumor suppressors known so far (e.g., BRAF and c-MYC vs p53 and PTEN) are in fact proteins. They have almost completely monopolized in vivo cancer modeling,^91^ and they are the targets of most of the current anticancer therapies, using synthetic small molecules^92^ or even other proteins (e.g., antibodies^93^). Considering the profound and vast knowledge on protein medicinal chemistry that we have accumulated, the recent discovery that several ncRNAs can be translated into peptides was good news to many. Even more recently, additional open reading frames (ORFs) have been found to reside within the 5′ untranslated region (UTR) of hundreds of mRNAs. These “upstream ORFs (uORFs)” are short and contribute to regulating the translation of the longer coding sequences (CDSs) located downstream.^94–98^

With this mindboggling complexity in mind, in this review we aim to provide an overview of cases in which the categorization of “coding gene” vs “non-coding gene” is outdated, since the same gene produces coding and non-coding elements.^99^ We also discuss the implications of this parsimonious usage of genetic units, in terms of independent or interdependent regulation of expression and concordant or discordant biological output in cancer. In addition, we point out the methodological advancements that are required to study bifunctional genes, with particular emphasis on the importance of in vivo modeling. Finally, we highlight the opportunities and challenges associated with the therapeutic implications of bifunctional genes in the development of anticancer therapies.

## Bifunctional genomic loci express mRNAs and ncRNAs

**Fig. 1:**
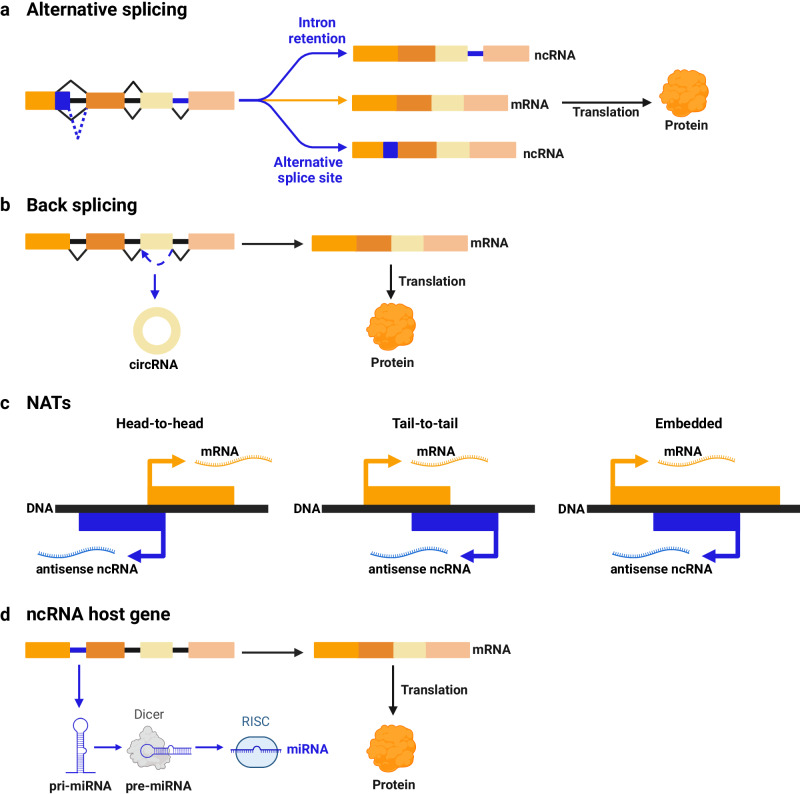
Bifunctional genomic loci express mRNAs and ncRNAs. Genomic loci are defined bifunctional when they can be considered both coding (orange) and non-coding (blue), because they express both an mRNA and a ncRNA, through one of the following mechanisms. **a** The ncRNA is the product of alternative splicing, for example through the retention of introns or the choice of an alternative splice site. **b** In back-splicing, the joining of an upstream 3′ splice acceptor with a 5′ downstream splice donor leads to the production of a non-coding circRNA. **c** NATs are non-coding RNA molecules transcribed from the opposite DNA strand. According to the degree of overlap with sense coding mRNAs, NATs are defined as head-to-head (the overlap is in the 5′ region, left), tail-to-tail (the overlap is in the 3′ region, middle) or embedded (the overlap is complete, right). **d** Exons compose the mRNA that is translated into a protein, while introns compose a non-coding RNA such as a miRNA. The ncRNA is called “intragenic”, while the coding gene in which it resides is called “host gene”.

There are genomic loci that can be rightfully considered both coding and non-coding because they express both an mRNA and a ncRNA, in a mutually exclusive or coexisting fashion (Fig. 1). Such genomic loci are defined “hybrid” or “bifunctional”, and representative cancer-relevant examples are described below.

### Expression of an mRNA and a ncRNA depending on the exons spliced together

Alternative splicing (AS) is one of the mechanisms through which cells express more than one transcript from the same genomic locus, thereby enriching their transcriptome (and proteome). Alternative splicing comes in five main flavors (exon skipping, alternative 5′ splice site, alternative 3′ splice site, mutually exclusive exons, intron retention) and it can occur in conjunction with the use of alternative transcription start sites (promoters), or alternative polyadenylation sites.^100–102^

Several examples have been reported of genomic loci that undergo alternative splicing and end up expressing a coding transcript and a non-coding transcript with a role in cancer (Fig. 1a).^103^ Two of such examples are described below.

The *Steroid Receptor RNA Activator 1* (*SRA1*) gene was the first bifunctional gene to be characterized. It expresses Steroid Receptor co-Activator Protein (SRAP), an oncogenic protein that acts as positive transcriptional regulator of steroid receptors. In breast cancer SRAP is overexpressed and associated with worse prognosis,^104^ while in prostate cancer it has been shown to potentiate the activity of the androgen receptor.^105^ Due to intron 1 retention,^106^ the *SRA1* gene expresses *lincRNA-SRA* as well. Oncogenic *lincRNA-SRA* is overexpressed in melanoma. In this context, it induces cell proliferation, migration, invasion, epithelial to mesenchymal transition (EMT), as well as metastasis in a xenograft model.^107^ However, the molecular mechanism(s) of its action remain to be elucidated.

The *PPP1R10* gene expresses Protein Phosphatase-1 (PP-1) Nuclear Targeting Subunit (PNUTS), a ubiquitous nuclear protein that binds to PP-1. In turn, PP-1 is a serine/threonine phosphatase mainly involved in chromosome decondensation at mitosis exit.^108^ PNUTS is a positive modulator of PP-1 activity, thereby favoring the re-entry into interphase.^109^ It is also involved in DNA damage repair.^110^ In the context of breast cancer, *PPP1R10* gene expresses *lncRNA-PNUTS* as well, due to an alternative 3′ splice site that is located in exon 12 and breaks the ORF encoding PNUTS protein. *lncRNA-PNUTS* expression is under the regulation of TGF-β: through AKT2-dependent phosphorylation, TGF-β causes the release of hnRNP E1 splicing repressor from a TGF-β Activated Translational (BAT) element that is positioned at the alternative splice site. In this way, alternative splicing can occur and *lncRNA-PNUTS* is expressed. In turn, *lncRNA-PNUTS* acts as an effector of TGF-β-induced EMT, because it sponges ZEB1-targeting miR-205. *lncRNA-PNUTS* silencing is in fact associated with decreased tumor initiation and metastasis in a xenograft model.^111^

Among common mechanisms through which coding and non-coding transcripts are expressed from the same genomic locus, there is also back-splicing (Fig. 1b). There are several examples of genomic loci expressing a linear coding mRNA and a circular non-coding RNA with roles in cancer.^84^

Specifically, we have attributed an oncogenic role to circular Pokémon (circPOK) in the context of mesenchymal tumors. circPOK is an EcircRNA generated from the *Zbtb7a* gene through back-splicing of exon 2. It promotes tumorigenesis by binding to the complex formed by InterLeukin enhancer binding Factor 2 and 3 (ILF2/3), hence sustaining the transcription/stability of multiple mRNAs encoding interleukins and angiogenic factors.^112^ Interestingly, the linear transcript expressed from *Zbtb7a* gene encodes Zbtb7a/Pokémon/Lrf, a transcriptional repressor that acts as a tumor suppressor of mesenchymal tumorigenesis, by promoting the differentiation of mesenchymal stem cells.^113^ The discordant functions of the linear coding transcript vs the circular non-coding transcript are confirmed by the opposite trend observed in their expression levels when mesenchymal tumors are compared to normal tissues: Pokémon levels are lower, while circPOK levels are higher. The decrease in Pokémon levels is due to enhanced post-transcriptional regulation by oncogenic miRNAs,^112^ while the mechanism behind the increase in circPOK remains to be established.

### Expression of a sense mRNA and an antisense ncRNA

In cancer, there are quite a few examples of genomic loci that are transcribed from both DNA strands and end up expressing a sense coding mRNA and an antisense non-coding RNA, i.e., an NAT (Fig. 1c).^65,66^ Two of such examples are described below.

As an example of *cis*-NAT, we highlight the oncogenic *ZEB1-AS1*. Zinc finger E-box-Binding homeobox 1 (ZEB1) is an oncogenic transcription factor overexpressed in many epithelial tumor types. ZEB1 is a master regulator of EMT, promoting migration/invasion in vitro and metastasis in vivo. It also confers resistance to chemotherapy.^114^ *ZEB-AS1* is transcribed in antisense orientation from the promoter region of *ZEB1* and in turn is involved in a positive feedback loop, sustaining ZEB1 expression at two levels. It promotes *ZEB1* transcription epigenetically. This is because it can recruit Mixed Lineage Leukemia 1 (MLL1) histone methyltransferase on the *ZEB1* promoter, therefore ensuring that H3 histone gets methylated at K4 (H3K4me3) and that DNA is accessible for transcription.^115^ In addition, *ZEB-AS1* sponges several anti-EMT miRNAs, including miR-200 family and miR-205, that in turn target *ZEB1*.^116,117^

As an example of *trans*-NAT, we mention the tumor-suppressive *CDR1as*. *Cerebellum Degeneration-Related antigen 1* (*CDR1*) is a low-expressed, poorly-characterized one-exon gene. *LINC00632* is a 5-exon lincRNA transcribed in antisense orientation compared to *CDR1*. *CDR1as* is generated by back-splicing of the 5th exon of *LINC00632*, which fully overlaps with *CDR1*.^118^ *CDR1as* is highly expressed in the brain, where it belongs to a sophisticated network that tightly regulates miR-7 level/activity and is involved in correct embryonic development.^119–124^ Conversely, *CDR1as* is expressed at low levels in all other tissues, except for melanocytes that share neural crest origin with the brain.^125^ In cancer, *CDR1as* is reported as downregulated both in gliomas and in melanoma, and it has been implicated in the regulation of protein stability and function.^126^ In gliomas, it stabilizes p53 by binding to it, hence preventing the binding of MDM2 and the consequent proteasome-dependent degradation.^127^ In melanoma, *CDR1as* inactivates the pro-metastatic IGF2BP3 protein by sponging it.^128^

### Expression of an mRNA from exons and of a ncRNA from introns of the same pre-mRNA

Bifunctional genomic loci exist where exons compose the mRNA that is translated into a protein, while introns compose a ncRNA. Such ncRNAs are called “intragenic” and the coding genes in which they reside are referred to as “host genes”.^129^

This arrangement is very common with miRNAs (Fig. 1d). Hundreds of miRNA genes are in fact located within introns of protein-coding genes. They can be transcribed from their own promoters as independent transcriptional units, but more commonly transcription starts from the promoter of the host gene and produces a bifunctional pre-mRNA: exons are spliced together into the mRNA, while the intronic pri-miRNA is further processed into mature miRNA(s). Consequently, miRNA(s) and host gene are co-expressed and co-involved in regulatory circuits.^130–132^

miR-106~25 cluster of miRNAs is composed of miR-106b, miR-93 and miR-25. It is located in the 13th intron of *Mini-Chromosome Maintenance protein 7* (*MCM7*) gene and is produced through pre-mRNA splicing. Both the miRNA cluster and the protein are aberrantly overexpressed in prostate cancer. MCM7 protein promotes the initiation of genome replication, while miR-106b~25 is responsible for the decrease of PTEN levels by binding to 3′UTR of *PTEN*. By generating a vector that drives the expression of *MCM7* exons as well as intron 13 (PIG/*MCM7i13*), we demonstrated that MCM7 protein and the miR-106~25 cluster can transform mouse embryonic fibroblasts in vitro. We then moved into the in vivo setting.

**Fig. 2:**
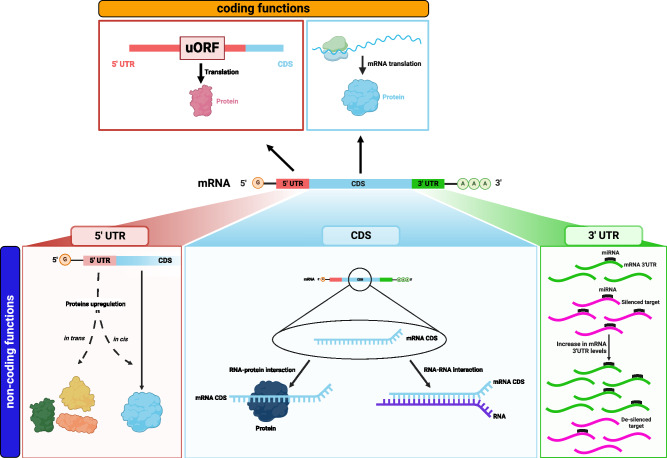
Bifunctional mRNAs exert coding functions and non-coding functions. In mRNA molecules (middle) three regions can be identified: the 5′UTR (red), the CDS (light blue) and the 3′UTR (green). (Top, orange) mRNAs are primarily protein-coding RNA molecules: they carry a primary ORF (the CDS), and they may also present a short uORF in the 5′UTR. (Bottom, blue) non-canonical non-coding functions have been attributed to mRNAs. The 5′UTR can exert non-coding functions *in cis* or *in trans*, by interacting with proteins. The CDS can be involved in non-coding RNA–protein or RNA–RNA interactions. The 3′UTR can exert non-coding functions within the concept of ceRNAs: due to MREs, 3′UTRs can sponge miRNAs, leading to the de-silencing of ceRNA partners that share MREs for the same miRNAs.

The *MCM7i13* construct was placed under the control of the prostate-specific rat Probasin promoter ARR_2_PB (*Pb*/*MCM7i13*). Nine transgenic lines were analyzed and clustered into low expressor (LE), medium expressor (ME) and high expressor (HE), according to the degree of overexpression of MCM7, miR-106b, miR-93 and miR-25. By analyzing a cohort of 1-year-old transgenic mice belonging to the various lines, we observed that the dorsolateral prostates (DLPs) displayed multifocal lesions whose severity correlated with transgene expression levels: while LE mice showed just hyperplasia, ME and even more HE mice showed typical histological features of prostatic intraepithelial neoplasia (PIN). In the DLPs of HE transgenic mice aberrant proliferation was confirmed by increased number of Ki67-positive cells, while the downregulation of *Pten* mRNA levels together with hyperactivation of Akt signaling was consistent with the activity of miR-106~25 as a *Pten*-targeting cluster. These data confirm that the MCM7 protein and the miR-106~25 cluster are 2 hits that can initiate prostate tumorigenesis in a tissue-specific mouse model. Interestingly, *Pb*/*MCM7* transgenic mice were generated as well. However, no signs of PIN were visible up to 1 year of age. This further confirms that MCM7 protein cannot initiate prostate tumorigenesis alone and that miR-106~25 non-coding cluster is indispensable.^133^

In conclusion, all these specific examples concur to highlight the complexity of gene expression and demonstrate that a single genomic locus can exert both coding functions, through the transcription of an mRNA and the synthesis of the corresponding protein, and non-coding functions, by expressing a ncRNA, with important biological outcomes.

## Bifunctional mRNAs exert non-coding functions as well

mRNAs are protein-coding RNAs, as their fate is to be exported from the nucleus to the cytoplasm, where they are translated into proteins. However, literature provides extensive evidence that mRNAs exert non-coding functions as well.

Here, we present examples of “bifunctional mRNAs” and of the role that they play in cancer because of the non-coding activities of their 5′UTR, CDS and 3′UTR (Fig. 2).

### The 5′UTR exerts non-coding functions

Two clear examples of 5′UTRs with non-coding functions are represented by the *c-Myc* and *Vascular Endothelial Growth Factor* (*VEGF*) genes. One (*c-MYC* P0 5′UTR) behaves as a tumor suppressor and the other (*VEGF* 5′UTR) as an oncogene.

The proto-oncogene *c-MYC* promotes cell proliferation and tumorigenesis. Although the most expressed *c-MYC* mRNAs are transcribed from the P1 and P2 transcription start sites, 5% of *c-MYC* transcripts are expressed from the upstream P0 transcription start site. P0 transcript is under the control of its own promoter and differs from the predominant P1 and P2 transcripts because of an extended 5′UTR (~640-nt extension). Blume et al.^134^ discovered that the overexpression of P0 5′UTR strongly decreases the anchorage-independent growth of HeLa cells in vitro, and their ability to form tumors when xenografted in nude mice. Digging into the molecular mechanism underlying this phenomenon, they found that P0 5′UTR overexpression is associated with the upregulation of the c-MYC2 (p64) protein, which is translated from the P2 transcript. Such upregulation results in increased apoptotic cell death, likely a failsafe mechanism triggered in the presence of excessive oncogenic signaling.^135^ Since no increase was observed in the level of the endogenous P2 transcript, the authors hypothesized that P0 5′UTR might work *in cis* by affecting the translation of the abovementioned c-MYC2 protein isoform, either directly through P0–P2 RNA interactions or indirectly through RBPs. It also remains to be elucidated which transcription factors control transcription from P0 vs P1/2 start site and how the choice is regulated in physiological and pathological conditions.

VEGF plays a critical role in tumorigenesis through promotion of neoangiogenesis.^136^ However, the susceptibility of HCT116 colon cancer cells to chemotherapeutic agents (5-fluorouracil, etoposide and doxorubicin) is not fully rescued by recombinant VEGF protein, which raises the possibility that *VEGF* mRNA exerts coding-independent functions. Masuda et al.^137^ unveiled that the overexpression of *VEGF* 5′UTR increases the anchorage-independent growth of HCT116 cells in vitro, and their ability to form tumors when xenografted in athymic nude mice. These tumors were profiled by microarray analysis, showing the upregulation of anti-apoptotic genes and downregulation of pro-apoptotic genes. Specifically, the authors observed a decrease in IFNα/STAT1-dependent pro-apoptotic signaling pathway, which resulted in decreased sensitivity of xenografted tumors to 5-fluorouracil, a chemotherapeutic agent that is known to elicit an IFNα/STAT1-dependent pro-apoptotic response. The tumor-promoting function was mapped to a 270 nt-long region located between 475 nt and 745 nt of *VEGF* 5′UTR and persisted when cells were treated with the translation inhibitor cycloheximide, which proves that it is not related to protein synthesis. However, the exact molecular mechanism still needs to be elucidated. Considering the wide impact on transcriptome and the strong biological effects, the authors speculate that *VEGF* 5′UTR works *in trans* as a regulatory RNA that affects the expression/functions of a wide network of target proteins.^137^

### The CDS exerts non-coding functions

The CDS is the region of the mRNA whose fate is to be translated, as its nucleotide sequence instructs the amino acid sequence of the protein, and that is why mRNAs are transcripts that primarily exert a coding function. However, there are cancer-relevant examples by which the CDS exerts a non-coding function as well.

The p53 transcription factor is a tumor suppressor protein that prevents cancerous transformation, mainly acting as a keeper of genome integrity.^138^ Under normal conditions p53 is kept inactive, while under stress conditions it gets rapidly activated. This is possible due to the tight control exerted by the MDM2 protein on p53. MDM2 binds to p53 and, functioning as an E3 ligase, triggers the ubiquitination of p53 C-terminal domain, and consequent proteasome-mediated degradation in the cytoplasm. Under stress conditions, MDM2 quickly releases its hold, so that p53 level increases, gets localized to the nucleus, and unleashes its activity as a transcription factor. Consequently, cells stop cycling and either repair the damage or die by apoptosis. Once damage is repaired, multiple negative feedback loops place p53 back under MDM2 control, so that the permanent blockage of cell functions is avoided.^139^

Extensive literature has unveiled the mechanisms through which p53 level increases in response to stress. DNA damage induces multiple post-translational modifications of p53 protein (phosphorylation and acetylation) that weaken its interaction with MDM2. It also induces p14/ARF that inactivates MDM2 by sequestering it into the nucleolus. Finally, relevant to the main topic of this review, the DNA damage sensor ATM phosphorylates MDM2 at Ser395. This induces a conformational change in the protein and favors its interaction with a specific sequence within the CDS of *p53* mRNA. Because of this interaction, MDM2 switches from a negative to a positive regulator of p53: it cannot function as E3 ligase any longer, and it rather promotes *p53* mRNA translation, contributing to the increased levels of p53 protein.^140–142^ Noteworthily, the BOX-I domain, which is the domain used by p53 protein to interact with MDM2 protein, is translated from the sequence used by *p53* mRNA to interact with MDM2 protein. Furthermore, the BOX-I domain is the most conserved region of p53 protein and has co-evolved with MDM2 protein. This attests that the coding and the non-coding functions of *p53* mRNA are equally important to ensure the fine regulation of p53 activation/deactivation.^143^

In normal somatic cells, progressive telomere shortening along with cell divisions results in replicative senescence. Conversely, cancer cells aberrantly express the TElomerase Reverse Transcriptase (TERT) enzyme that forms a ribonucleoprotein (RNP) with Telomerase RNA (TR) and uses it as a template to extend telomere length, counteracting telomere erosion. Since TR level exceeds TERT level, TERT-independent functions have been postulated for TR. Ivanyi-Nagy et al.^144^ used a pull-down approach to map the RNA interactome of TR. Among the candidates further studied, *HIST1H1C*, the mRNA of H1.2 linker histone subtype, was found to exert a non-coding activity through its CDS. Within *HIST1H1C* CDS, nucleotides 334–348 form an RNA duplex with TR and were named Telomerase RNA InterActing Genetic Element (TRIAGE). The direct interaction of TRIAGE with TR does not affect TERT enzymatic activity, and yet it has a negative impact on telomere elongation. The authors hypothesize that, by base pairing with TR, TRIAGE sponges TERT RNP away from telomeres, impairing their elongation.

### The 3′UTR exerts non-coding functions

It is well established that 3′UTRs exert regulatory functions and have an impact on tumorigenesis. For example, we have recently reported in a zebrafish model of melanoma that the presence of the 3′UTR impacts on BRAFV600E-driven tumorigenesis. The strong melanoma driver effect of the CDS of reference BRAFV600E (BRAFV600E-ref) is in fact suppressed in the presence of the corresponding 3′UTR.^145^

3′UTRs are post-transcriptional regulators of gene expression, and they exert both *in cis* and *in trans* functions. *In cis* functions are mainly coding-related, as they involve the regulation of stability, localization, and translation of the mRNA itself.^146,147^ By contrast, the mechanism through which 3′UTRs exert their non-coding function *in trans* falls within the concept of mRNAs as sponges for miRNAs, i.e., as ceRNAs. Hundreds of papers about this topic have been published in the last decade, most of which are in the context of cancer. Indeed, when 3′UTR-dependent functions are at play, the overexpression of an oncogenic mRNA or the downregulation of a tumor-suppressive mRNA promotes cancer initiation and/or progression not only because of the encoded protein, but also because of the ceRNA activity of its 3′UTR. Three examples are described below.

In breast cancer the *CXCR4* mRNA promotes metastasis not only through its protein, the CXCR4 chemokine receptor, but also through the *CXCR4* 3′UTR that sponges the tumor-suppressive miR-146a, thus leading to the upregulation of TRAF6 and EGFR, two oncoproteins that activate the NF-κB pathway.^148^

The *PTEN* mRNA is renowned to suppress oncogenic PI3K/AKT signaling pathway through the PTEN protein, a phosphatase that dephosphorylates phosphatidylinositol-3,4,5-trisphosphate (PIP3) to phosphatidylinositol-4,5-biphosphate (PIP2) and in so doing prevents downstream AKT activation.^149^ However, the *PTEN* mRNA exerts a tumor-suppressive function also by sponging oncogenic miRNAs, and hence by sustaining the expression of tumor-suppressive ceRNA partners involved in other signaling pathways.^70,150,151^ In turn, the ceRNA network of mRNAs that sustains the expression of the *PTEN* mRNA, and hence the inhibition of the PI3K/AKT signaling pathway, is even more extended^34,150–155^ and fatally gets heavily affected by the widespread 3′UTR shortening that occurs in cancer.^156^

Quite interesting is also the case of the ZEB1 and ZEB2 transcription factors and master regulators of EMT (see “Expression of a sense mRNA and an antisense ncRNA” chapter above). In epithelial tumors, the ZEB1 protein is oncogenic because it promotes EMT not only directly (by repressing the transcription of miR-200 family), but also indirectly: it induced the transcription of *Integrin A1* mRNA, which in turn sponges the tumor-suppressive miR-181b away from *Adenylyl Cyclase 9* (*ADCY9*) mRNA. As a result, the levels of ADCY9 protein increase, as well as those of cyclic AMP, which favors metastatic dissemination.^157^ In melanoma *ZEB2* behaves as a tumor suppressor not only because ZEB2 protein activates MITF-dependent differentiation program,^158^ but also because *ZEB2* 3′UTR has *PTEN* mRNA as ceRNA partner.^151^

For further examples of specific mRNAs with ceRNA activity, please refer to Table 1. Examples of extended ceRNA networks that revolve around mRNAs are reported in.^153,159–161^

**Table 1:** List of mRNAs that exert ceRNA activity in human cancer due to their 3'UTR.

3'UTR	miRNA	DE-SILENCED mRNA	TUMOR TYPE	SPONGING ACTIVITY OUTPUT	IN VIVO MODELS	Ref #
			Non Small Cell Lung Cancer	Oncogenic	-	^214^
			Colorectal Cancer	Tumor-suppressive	xenograft	^215^
			Breast Cancer	Tumor-suppressive	xenograft	^216^
			Breast Cancer	Oncogenic	xenograft	^217^
			Breast Cancer	Oncogenic	xenograft	^217^
			Breast Cancer	Tumor-suppressive	xenograft	^218^
			Liver Cancer Stem Cells	Tumor-suppressive	-	^219^
			Breast Cancer	Tumor-suppressive	xenograft + patients	^220^
			Urothelial Carcinoma	Oncogenic	xenograft + patients	^221^
			Colorectal Cancer and Prostate Cancer	Tumor-suppressive	ref. ^151^	^150^
			**Breast Cancer**	**Oncogenic**	**-**	^148^
			Breast Cancer	Tumor-suppressive	-	^222^
			Breast Cancer	Oncogenic	chick embryo + rat	^223,224^
			Colorectal Cancer	Tumor-suppressive	-	^155^
			Ovarian Cancer	Oncogenic	xenograft + patients	^225^
			Breast Cancer	Tumor-suppressive	-	^226^
			**Lung Cancer (+713P, 307P, 344LN, 393LN, 393P, 412P, 344SQ, 344P, 531LN1, 531LN2, 531P1, and 531P2 murine cell lines)**	**Oncogenic**	**xenograft**	^157^
			Hepatocellular Carcinoma	Oncogenic	xenograft + patients	^227^
			Oral Squamous Cell Carcinoma	Oncogenic	patients	^228^
			Non Small Cell Lung Cancer	Oncogenic	patients	^229^
			Triple Negative Breast Cancer (+4T1 murine cell line)	Oncogenic	xenograft + patients	^230^
			Hepatocellular Carcinoma	Oncogenic	xenograft + patients	^231^
			Non Small Cell Lung Cancer	Oncogenic	-	^232^
			Ovarian Cancer	Oncogenic	-	^233^
			Ovarian Cancer	Oncogenic	-	^234^
			Breast Cancer	Tumor-suppressive	-	^235^
			Breast Cancer	Tumor-suppressive	xenograft	^236^
			Breast Cancer	Tumor-suppressive	xenograft	^237^
			Hepatocellular Carcinoma	Tumor-suppressive	patients	^238^
			Prostate Cancer	Tumor-suppressive	-	^154^
			Colorectal Cancer	Tumor-suppressive	xenograft + patients	^239^
			**Colorectal Cancer and Prostate Cancer**	**Tumor-suppressive**	**-**	^150^
			Hepatocellular Carcinoma	Oncogenic	transgenic mice + patients	^240^
			Breast Cancer (+4T1 murine cell line)	Tumor-suppressive	xenograft + transgenic mice	^152^
			Breast Cancer (+4T1 murine cell line)	Tumor-suppressive	xenograft + transgenic mice	^152^
			Breast Cancer and Colorectal Cancer	Oncogenic	xenograft	^241^
			Hepatocellular Carcinoma	Oncogenic	-	^242^
			**Colorectal Cancer and Melanoma (+TB13602 murine cell line)**	**Tumor-suppressive**	**xenograft + transgenic mice**	^151^

Bold: examples described in the text

## Bifunctional ncRNAs exert coding functions as well

**Fig. 3:**
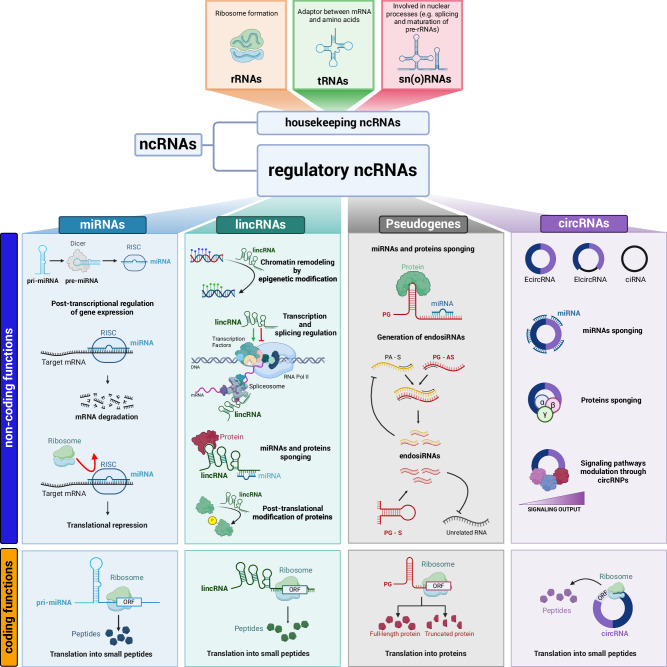
Bifunctional ncRNAs exert non-coding functions and coding functions. ncRNAs can be divided into two groups: housekeeping ncRNAs (upper panels) and regulatory ncRNAs (lower panels). Among housekeeping ncRNAs there are rRNAs, tRNAs, snRNAs and snoRNAs. Regulatory ncRNAs are further divided according to their length. Among short ncRNAs (< 200 nt) there are miRNAs (light blue). Long ncRNAs (lncRNAs, ≥ 500 nt) include lincRNAs (light green), pseudogenic RNAs (PGs, light gray), NATs (see Fig. 1c), and circRNAs (light purple). The primary function of miRNAs is non-coding (blue) and relates to post-transcriptional regulation of gene expression: mRNA degradation or translational repression mediated by the RISC complex. Among non-coding functions of lincRNAs there are: chromatin remodeling by epigenetic modification, transcription and splicing regulation, sponging of miRNAs and proteins, post-translational modification of proteins. Non-coding functions of PGs can be parental gene (PA)-related or unrelated. They include sponging of miRNAs and proteins, and mRNA degradation through endosiRNAs. S sense strand, AS antisense strand. circRNAs can be composed by only exons (EcircRNAs), both exons and introns (EIcircRNAs), or only introns (ciRNAs). They exert non-coding functions by acting as sponges for miRNAs and proteins, and by forming circRNPs that modulate signaling pathways. These groups of regulatory ncRNAs can all exert non-canonical coding functions as well, because they can be translated into ncPEPs, i.e., small peptides or proteins (orange).

In the last years, polysome/ribosome profiling and mass spectroscopy analyses have provided evidence that, together with^162^ or alternatively to^163–165^ coding-independent activities, ncRNAs can carry ORFs that are translated into ncRNA-encoded peptides (ncPEPs).^166–168^ The ~100 ncPEPs characterized so far are mostly short (< 100 aa), but longer examples exist.^167,169–171^ Many of these ncPEPs have an impact in cancer, as described below (Fig. 3).

### Translation of pri-miRNAs

The presence of a short ORF has been demonstrated within the pri-miRNA of miR-200a and miR-200b, two miRNAs with tumor-suppressive activity that inhibit metastasis by blocking EMT (see “Expression of a sense mRNA and an antisense ncRNA” chapter and “The 3′UTR exerts non-coding functions” chapter above). When overexpressed in prostate cancer cells, these pri-miRNA-encoded peptides, called miPEP-200a and miPEP-200b, have been shown to inhibit migration and to impair EMT. However, the molecular mechanism of action and the potential synergy with the corresponding miRNAs remain to be established.^172^

### Translation of lincRNAs

Recent reports indicate that several lincRNAs contain ORFs that encode peptides and attest the importance of this feature in the development and progression of cancer.^59^ Two examples are described below.

*LINC00665* is a lincRNA aberrantly expressed in more than 15 cancer types. It is involved in several signaling pathways (Wnt/β-Catenin, TGF-β, NF-κB, PI3K/AKT, and MAPK) and has attracted attention as a diagnostic and prognostic marker. In most of the cases, *LINC00665* is an oncogenic lincRNA that exerts non-coding activities by working as ceRNA for more than 20 miRNAs, as well as by binding to chromatin remodeling factors and transcriptional regulators.^163^ By contrast, in triple-negative breast cancer (TNBC), it acts as a tumor-suppressive lincRNA that expresses CIP2A-BP. This is a 52-aa peptide that in vitro inhibits migration and invasion of TNBC cells. Mechanistically, CIP2A-BP impairs the PI3K/AKT signaling pathway by binding to Cellular Inhibitor of PP2A (CIP2A). Consequently, CIP2A no longer binds to PP2A phosphatase, and in turn PP2A inactivates AKT by dephosphorylation at Thr308 and Ser473 residues. Conversely, the EMT-promoting TGF-β signaling pathway actively downregulates CIP2A-BP. Specifically, the TGF-β effector SMAD4 upregulates *4E-BP1* transcription. In turn, 4E-BP1 binds to eIF4F and inhibits translation of many proteins, including CIP2A-BP.^173^

To study the role of the CIP2A-BP peptide in tumorigenesis in vivo, a knock-in (KI) mouse model was developed. Specifically, homologous recombination was used to knock in *CIP2A-BP* ORF, preceded by a loxed STOP codon, into the *ROSA26 (R26)* locus, which ensures robust transcription under the *R26* promoter. KI mice were then crossed with *CMV-Cre* mice so that homozygous *CIP2A-BP*^*+/+*^ mice were obtained, which show constitutive and ubiquitous CIP2A-BP translation. Finally, *CIP2A-BP*^*+/+*^ mice were further crossed with breast cancer-prone *MMTV-PyMT* mice, obtaining *MMTV-PyMT;CIP2A-BP*^*+/+*^ mice. *MMTV-PyMT* is the most widely used genetically engineered model (GEM) of breast cancer in the mouse. In this model, Murine Mammary Tumor Virus (MMTV) Long Terminal Repeat (LTR) promoter restricts the expression of PolYoma Middle T antigen (PyMT) to the mammary epithelium. This leads to the formation of multifocal primary tumors that have high tendency to metastasize to the lung.^174^ In accordance with in vitro data, the combined expression of PyMT and CIP2A-BP in the mammary epithelium, which characterizes *MMTV-PyMT;CIP2A-BP*^*+/+*^ mice, is associated with decreased AKT phosphorylation in primary tumors and decreased number of metastatic nodules in the lungs.^173^

Another interesting example is represented by *LINC00998*. In hepatocellular carcinoma (HCC), *LINC00998* is upregulated and is associated with worse prognosis. This lincRNA is under the transcriptional control by c-MYC and expresses a 59-aa peptide termed SMIM30, which shows oncogenic properties both in vitro and in xenograft mouse models. In fact, SMIM30 overexpression results in increased proliferation, migration and invasion. Mechanistically, SMIM30 localizes at the plasma membrane, where it binds to YES1, a member of SRC tyrosine kinase family. Binding to SMIM30 increases membrane anchoring of YES1 and facilitates downstream activation of oncogenic MAPK signaling pathway.^175^

Additional examples of lincRNA-encoded peptides are listed in Table 2 (upper).

**Table 2:** List of lincRNAs and PGs that are translated into peptides/proteins with a role in human cancer.

lincRNA	ENCODED PEPTIDE	TUMOR TYPE	PEPTIDE EXPRESSION LEVEL	PEPTIDE ACTIVITY OUTPUT	PEPTIDE BIOLOGICAL ACTIVITY	IN VIVO MODELS	Ref #
LINC00266-1	RBRP	Colorectal cancer		Oncogenic	Binding to the m6A reader IGF2BP1. This results in increased c-Myc expression.	xenograft + patients	^243^
LINC00278	YY1BM	Esophageal squamous cell carcinoma		Tumor-suppressive	Separation of YY1 from AR. This results in eEF2k downregulation.	xenograft + patients	^244^
**LINC00665**	**CIP2A-BP**	Triple negative breast cancer		Tumor-suppressive	Binding to CIP2A. This results in increased dephosphorylation of AKT by PP2A, hence decreased signaling through the PI3K/AKT pathway.	xenograft + transgenic mice + patients	^173^
LINC00675	FORCP	Colorectal cancer		Tumor-suppressive	Promotion of cancer cell apoptosis and suppression of tumorigenesis, possibly through the regulation of BRI3BP function.	xenograft	^245^
LINC00908	ASRPS	Triple negative breast cancer		Tumor-suppressive	Impairment of STAT3 phosphorylation. This results in decreased VEGF expression.	xenograft + transgenic mice + patients	^246^
**LINC00998**	**SMIM30**	Hepatocellular carcinoma		Oncogenic	Binding to YES1. This results in increased signaling through the MAPK pathway.	xenograft + patients	^175^
**PG**	**ENCODED PEPTIDE**	**TUMOR TYPE**	**PEPTIDE EXPRESSION LEVEL**	**PEPTIDE ACTIVITY OUTPUT**	**PEPTIDE BIOLOGICAL ACTIVITY**	**IN VIVO MODELS**	
BRAF pseudogene	BRAF pseudogene peptide	Thyroid cancer (+CHO hamster cell line and NIH3T3 murine cell line)		Oncogenic	Promotion of signaling through the MAPK pathway. This leads to NIH3T3 cell transformation.	xenograft + patients	^247^
CRIPTO3	CRIPTO3 protein	Cancer (+F9 murine cells (cripto–/–, Nodal+))		Oncogenic	Activation of Nodal signaling pathway.	patients	^248^
MAPK6P4	P4-135aa	Glioblastoma		Oncogenic	Phosphorylation and stabilization of KLF15.	xenograft + patients	^249^
**NANOGP8**	**NANOGP8 protein**	Prostate cancer		Oncogenic	Cancer initiation in combination with c-MYC.	transgenic mice	^192^
NA88-A	NA88-A peptide	Melanoma		Tumor-suppressive	Antigenic peptide that is recognized by CD8+ T cells.	–	^250^
OCT4-PG1	OCT4-PG1 protein	Chronic myeloid leukemia		Oncogenic	Alteration of multidrug resistance phenotype by directly interacting with OCT4, SOX2, and NANOG and indirectly with ABC transporters.	–	^251^
STK24P1	P1-121aa	Glioblastoma		Oncogenic	Phosphorylation and stabilization of ELF2. This results in increased VEGFR2 and VE-cadherin expression.	xenograft + patients	^252^

Bold: examples described in the text

### Translation of PGs

It was estimated that dozens of pseudogenic proteins exist, and some can be longer than 100 aa.^176^ They are mostly expressed from processed pseudogenes, therefore they do not share the same promoter with their parental genes and, although they might be highly homologous in sequence, they get expressed in distinct tissues, subcellular compartments, or pathophysiological conditions. Pseudogenic proteins specifically expressed in cancer might also carry mutations that further alter their functioning.^68,177^

NANOGP8 is an example of pseudogenic protein with an oncogenic role. Together with LIN28, OCT4 and SOX2, NANOG forms a core network of transcription factors that regulate self-renewal of stem cells. Cancer cells often re-acquire stem-like properties and one of the mechanisms is the aberrant expression of NANOGP8, the NANOG-like protein translated from *NANOGP8* processed pseudogene.^178,179^ NANOGP8 is detectable in various cancer types, including prostate cancer and cancers of the gastrointestinal tract, and its levels are particularly high in the cancer stem cell subpopulation.^180–185^ Accordingly, NANOGP8 displays oncogenic properties: it promotes clonogenicity, survival, proliferation, migration, anchorage-independent growth and resistance to anticancer drugs, both in vitro and in xenograft models.^186–190^ Furthermore, a NANOGP8 signature is associated with worse prognosis.^191^

Importantly, a transgenic mouse model was developed to study the contribution of NANPOGP8 to prostate tumorigenesis in vivo. The ORF of *NANOGP8* was placed under the control of the prostate-specific Probasin promoter mentioned above (*Pb*/*NANOGP8*). After monitoring a cohort of transgenic animals for up to 2 years, the authors did not observe histological evidence of hyperplasia or PIN. However, when they crossed *Pb*/*NANOGP8* transgenic mice with *Pb*/*c-MYC* transgenic mice, they observed an exacerbated phenotype, with thicker epithelial layers containing more atypical cells. These results indicate that NANOGP8 is not sufficient to initiate prostate cancer, although it cooperates with c-MYC.^192^ Interestingly, similar results were obtained in additional transgenic models. When NANOGP8 was overexpressed in epithelial organs through the *Cytokeratin 14 (K14)* promoter, no spontaneous tumor development was detected even after a prolonged time of observation.^193^ Analogously, the targeted overexpression of Nanog in the mammary gland was not sufficient to induce mammary tumors. However, in the presence of the concomitant overexpression of Wnt-1, Nanog contributed to the decreased mouse survival due to highly enhanced metastatic burden.^194^ In summary, NANOGP8 overexpression/inhibition causes a strong increase/decrease in prostate cancer cell line growth, when xenografted into immunodeficient mice,^180–182,186,191,195^ but NANOGP8 overexpression is unable to initiate prostate tumorigenesis in a transgenic mouse.^192,193^ These results show the importance of GEMs to assess the specific role played by the gene of interest in each phase of cancer development, from initiation to metastasis.

Additional examples of PG-encoded peptides are listed in Table 2 (lower).

### Translation of circRNAs

About 1% of circRNAs are translated.^81^ This mainly applies to circRNAs composed only by exons (EcircRNAs), as they localize in the cytoplasm.^196^ Translation remains polysome-dependent, but, in the absence of 5′ cap and 3′ polyA tail, it is cap-independent. Three main mechanisms have been identified thus far. One relies on the presence of an Internal Ribosome Entry Site (IRES), a sequence that can directly recruit ribosomes to initiate translation. Another cap-independent translation mechanism is mediated by *N*^6^-methyladenosine (m^6^A) residues. It has been reported that at least 13% of circRNAs carry m^6^A modifications and that just one modification per circRNA is sufficient to promote the recruitment of the m^6^A reader YTHDF3, as well as eIF3A and eIF4s (A, B, G2) translation initiation factors. Finally, rolling translation occurs when the circRNA harbors the ATG, but not the stop codon. Translation is carried on in an infinite circle, until it is interrupted through a mechanism termed –1 Programmed Ribosomal Frameshifting (–1 PRF)-mediated Out-of-frame Stop Codon (OSC).^197–199^

circRNA translation is a field that has just started to be explored. Approaches for the detection of circRNA-encoded peptides^200^ and tools for their study^201^ have been developed. Yet, they require further refinement to exclude the unintended detection of peptides produced by the linear counterparts.^200^ Nevertheless, solid experimental evidence is accumulating about circRNA-encoded peptides that play key roles in cancer.^202–204^ Two examples are described below.

In glioblastoma multiforme (GBM), AKT3-174aa ncPEP is encoded by *hsa_circ_0017250/circAKT3*, a circRNA that comprises exons 3–7 of the *AKT3* gene. Together with AKT1 and 2, AKT3 is an oncogenic kinase with a well-established role in the PI3K/AKT signaling pathway. Since they activate key downstream effectors of this pathway, AKTs are involved in many aspects of tumor initiation and progression, up to metastasis and drug resistance. They are also actively investigated as therapeutic targets.^205^ AKT3-174aa shows decreased expression levels in GBM tissues compared to adjacent normal brain tissues and displays oncosuppressive properties: when overexpressed, it decreases GBM cell proliferation in vitro and in xenograft models. It also increases sensitivity to radiation. Furthermore, higher AKT3-174aa levels are associated with better prognosis in patients. Mechanistically, AKT3-174aa competes with AKT3 for binding to phosphoinositide-dependent kinase-1 (PDK1). In so doing, it prevents AKT3 activation by phosphorylation on Thr308. Therefore, AKT3-174aa should be considered as a dominant-negative AKT3 isoform that restrains AKT3 activity through a negative feedback loop.^164^

In GBM and TNBC, FBXW7-185aa ncPEP is encoded by *hsa_circ_022705/circFBXW7*, a circRNA that comprises exons 3–4 of the *FBXW7* gene. FBXW7 is a tumor-suppressive E3 ubiquitin ligase that targets multiple oncogenic proteins, including c-MYC, for proteasome-dependent degradation.^206^ FBXW7-185aa shows decreased expression levels in cancer tissues compared to adjacent normal tissues and has tumor-suppressive properties: when overexpressed, it decreases cancer cell proliferation in vitro and in xenograft models. Furthermore, higher FBXW7-185aa levels are associated with better prognosis in patients. Mechanistically, FBXW7-185aa has a strong affinity for Ubiquitin Specific Protease 28 (USP28), a de-ubiquitinating enzyme that prevents c-MYC degradation. By binding to USP28, FBXW7-185aa impairs its binding to c-MYC, and hence enhances the proteasome-dependent degradation induced by FBXW7.^162,207^ Interestingly, in TNBC hsa_circ_022705 not only favors FBXW7 activity through FBXW7-185aa ncPEP, but also sustains *FBXW7* expression by sponging miR-197-3p.^162^

Additional examples of circRNA-encoded peptides are listed in Table 3.

**Table 3:** List of circRNAs that are translated into peptides with a role in human cancer.

circRNA	ENCODED PEPTIDE	TUMOR TYPE	PEPTIDE EXPRESSION LEVEL	PEPTIDE ACTIVITY OUTPUT	PEPTIDE BIOLOGICAL ACTIVITY	IN VIVO MODELS	Ref #
**circAKT3**	**AKT3-174aa**	Glioblastoma		Tumor-suppressive	Competitive binding to PDK1. This results in decreased AKT3 phosphorylation, hence decreased signaling through the PI3K/AKT pathway.	xenograft + patients	^164^
circASK1	ASK1‐272aa	Lung adenocarcinoma		Tumor-suppressive	Competitive binding to AKT1. In this way ASK1 is released from phosphorylation‐mediated inactivation.	patients	^253^
circAXIN1	AXIN1‐295aa	Gastric cancer		Oncogenic	Binding to APC. This prevents the interaction of APC with AXIN1, abolishing its inhibitory effect. As a result, signaling through the Wnt/β‐Catenin pathway increases.	xenograft + patients	^254^
circβ‐Catenin	β-Catenin-370aa	Non-small cell lung cancer		Oncogenic	Competitive binding to GSK3β, which prevents the GSK3β‐mediated degradation of β‐Catenin. This results in increased signaling through the Wnt/β‐Catenin pathway.	patients	^255^
		Liver cancer		Oncogenic		xenograft + patients	^256^
circCHEK	circCHEK1-246aa	Multiple myeloma		Oncogenic	Promotion of cell proliferation through chromosomal instability; enhancement of macrophage‐osteoclast differentiation.	xenograft	^257^
circDIDO1	DIDO1‐529aa	Gastric cancer		Tumor-suppressive	Promotion of the ubiquitin‐mediated degradation of PRDX2.	xenograft + patients	^258^
circE‐Cad	C‐E‐Cad	Glioblastoma		Oncogenic	Promotion of EGFR signaling.	xenograft + patients	^259^
circEIF6	EIF6‐224aa	Triple negative breast cancer		Oncogenic	Promotion of the MYH9/Wnt/β‐Catenin signaling pathway.	xenograft + patients	^260^
**circFBXW7**	**FBXW7-185aa**	Glioma		Tumor-suppressive	Binding to USP28. This favors the proteasome-dependent degradation of c‐Myc induced by FBXW7.	xenograft + patients	^207^
		Triple negative breast cancer				xenograft + patients	^162^
circFGFR1	circFGFR1p	Cancer		Tumor-suppressive	Negative regulation of FGFR1.	–	^261^
circFNDC3B	circFNDC3B‐218aa	Colorectal cancer		Tumor-suppressive	Inhibition of the Snail‐FBP‐EMT axis.	xenograft + patients	^262^
circGprc5a	circGprc5a-peptide	Bladder cancer		Oncogenic	Binding to Gprc5A. This results in increased signaling through the GPCR pathway.	patients	^263^
circHEATR5B	HEATR5B‐881aa	Glioblastoma		Tumor-suppressive	Mediation of the inhibitory effect of circHEATR5B.	xenograft + patients	^264^
circ‐HER2	HER2‐103	Triple negative breast cancer		Oncogenic	Binding to EGFR and HER3. This results in increased EGFR signaling.	xenograft + patients	^265^
circMAPK1	MAPK1‐109aa	Gastric cancer		Tumor-suppressive	Inhibition of MAPK1 phosphorylation. This results in decreased signaling through the MAPK pathway.	xenograft + patients	^266^
circMAPK14	circMAPK14‐175aa	Colorectal cancer		Tumor-suppressive	Competitive binding to MKK6. This results in decreased MAPK14 phosphorylation and leads to proteasome-dependent degradation of FOXC1.	xenograft + patients	^267^
circMAP3K4	circMAP3K4‐455aa	Hepatocellular carcinoma		Oncogenic	Alteration of the nuclear distribution of AIF.	xenograft + patients	^268^
circPLCE1	circPLCE1‐411	Colorectal carcinoma		Tumor-suppressive	Dissociation of the HSP90α/RPS3 complex, followed by proteasome-dependent degradation of RSP3. As a result, signaling through the NF‐κB pathway decreases.	xenograft + PDX + patients	^269^
circPPP1R12A	cPPP1R12A-73aa	Colon cancer		Oncogenic	Promotion of Hippo-YAP signaling pathway.	xenograft + patients	^270^
circSHPRH	SHPRH-146aa	Glioma		Tumor-suppressive	Promotion of proteasome-dependent degradation of PCNA.	xenograft + patients	^271^
circSMO	SMO‐193aa	Glioblastoma		Oncogenic	Promotion of HH signaling pathway (Shh/Gli1/FUS/SMO‐193aa/SMO).	xenograft + patients	^272^
circUBE4B	circUBE4B‐173aa	Esophageal squamous cell carcinoma		Oncogenic	Promotion of MAPK1 phosphorylation. This results in increased signaling through the MAPK pathway.	xenograft + patients	^273^
circ0000437	CORO1C‐47aa	Endometrial cancer		Tumor-suppressive	Inhibition of VEGF expression.	xenograft + patients	^274^
**circPINTexon2**	**PINT87aa**	Glioblastoma		Tumor-suppressive	Binding to PAF1. This results in the inhibition of the transcriptional elongation of multiple oncogenes.	xenograft + patients	^209^
ecircCUX1	p113	Neuroblastoma		Oncogenic	Formation of the p113/ZRF1/BRD4 transcriptional regulatory complex.	xenograft + patients	^275^

Bold: examples described in the text

## Concluding remarks

In the last few years, it has become evident that the complexity of our genome vastly exceeds the simple organization into genetic units that code for proteins. Proteins are certainly the building blocks of cellular and organismal structures, but hundreds of thousands of non-coding RNAs are also at play, and in turn peptides of various lengths can be translated outside the canonical CDSs. Therefore, the intricacy of the coding vs non-coding interplay is progressively unraveling with mindboggling scenarios. As a paradigmatic example of the blurry boundary between what is coding and what is non-coding, we highlight *long intergenic non-protein-coding RNA p53-induced transcript* (*LINC-PINT*), a nuclear lincRNA under the transcriptional control of p53. *LINC-PINT* exerts an oncosuppressive role, although the molecular mechanism varies from cancer type to cancer type. In colon cancer, it works as a long non-coding RNA: it binds to the Polycomb Repressive Complex 2 and in so doing it prevents the transcription of pro-proliferation and pro-survival genes.^208^ Conversely, in glioblastoma, although it does not show coding capabilities per se, *LINC-PINT* undergoes back-splicing of exon 2. The EcircRNA that is generated, named circPINTexon2, contains an IRES and is translated into an 87-aa peptide. In turn, it is the PINT87aa ncPEP to be endowed with a tumor-suppressive role: in the nucleus, it directly interacts with RNA Polymerase II-Associated factor 1 (PAF1) and inhibits the elongation of the primary transcript of several oncogenes.^209^

**Fig. 4:**
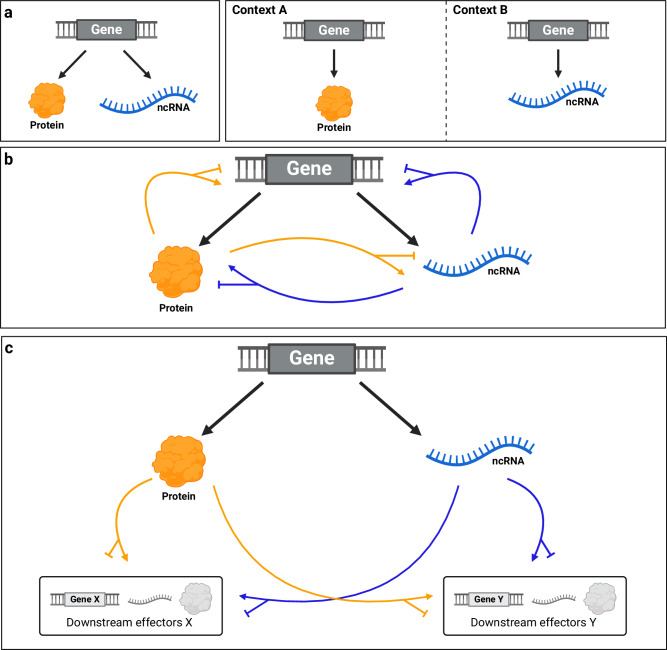
The complexity of expression and function of the coding and non-coding products derived from the same bifunctional gene. The coding product is represented as a generic orange protein and the non-coding product as a generic blue ncRNA. **a** Possible scenarios for gene expression. The protein and the ncRNA are expressed together in the same context (left), or separately in two different contexts (right). **b** Possible *in cis* regulatory mechanisms. The protein and the ncRNA positively or negatively regulate the gene from which they originate, or each other. **c** Possible *in trans* regulatory mechanisms. The protein and the ncRNA positively or negatively regulate downstream effectors (other genes, RNAs or proteins) that can be distinct or the same for both.

In spite of such a variegated use of coding and non-coding elements, in Fig. 4 we attempt to categorize the modalities of expression and function of the coding and non-coding products derived from the same bifunctional gene: the protein and the non-coding RNA are co-expressed (e.g., in TNBC, *hsa_circ_022705* is translated into FBXW7-185aa ncPEP and it also works as sponge for miR-197-3p^162^), or they are expressed in distinct tissues/physiopathologic conditions (e.g., *LINC00665* works as ceRNA for miRNAs in several cancer types, with the exception of TNBC where it is instead translated into CIP2A-BP peptide^163^) (Fig. 4a). The protein and the non-coding RNA act *in cis* on their own gene, regulating each other’s expression and/or activity (e.g., *ZEB-AS1 cis*-NAT sustains ZEB1 protein expression^115^) (Fig. 4b). Alternatively, they act *in trans* on other genes or their products (e.g., MCM7 promotes DNA replication and miR-106b~25 cluster downregulates PTEN expression^133^) (Fig. 4c). Finally, their activities can be concordant (e.g., p53 protein and the CDS that encodes it^140–142^) or discordant (e.g., AKT3 protein vs AKT3-174aa ncPEP^164^).

**Fig. 5:**
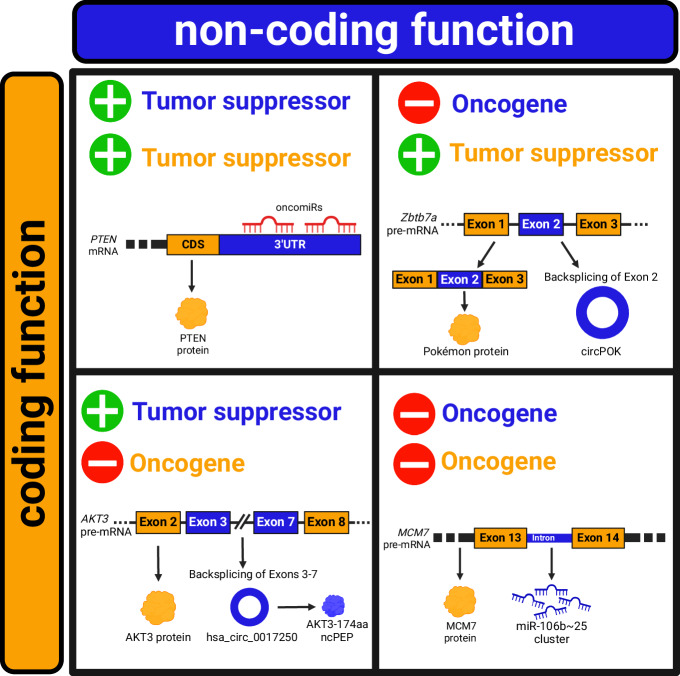
Concordant or discordant impact of the coding and non-coding products derived from the same bifunctional gene in cancer. Graphical representation of the possible combinations of cancer-related functions of coding (orange) and non-coding (blue) partners. Upper left panel: both the coding and the non-coding products have tumor-suppressive properties. *PTEN* mRNA encodes the tumor suppressor PTEN protein and, due to its 3′UTR, it can sponge oncogenic miRNAs. Upper right panel: the coding product is a tumor suppressor, while the non-coding product is an oncogene. *Zbtb7a* pre-mRNA undergoes canonical splicing and generates a mature mRNA that encodes the Pokémon protein with tumor suppressor properties; the same pre-mRNA also undergoes back-splicing of exon 2, leading to the formation of oncogenic circPOK. Bottom left panel: the coding product is an oncogene, while the non-coding product is a tumor suppressor. *AKT3* pre-mRNA is translated into AKT3 oncogenic protein, but exons 3–7 undergo back-splicing, leading to the production of a circRNA (*hsa_circ_0017250*) that exerts tumor-suppressive effects through its translation into the AKT3-174aa ncPEP. Bottom right panel: both the coding and the non-coding products have oncogenic properties. *MCM7* pre-mRNA is spliced to produce a mature mRNA that is translated into the oncogenic MCM7 protein; in addition, intron 13 hosts oncogenic miR-106b~25 cluster. From a therapeutic point of view, the optimal approach is to enhance/restore tumor-suppressive activities (green “plus” symbol) and/or, on the other hand, abolish/inhibit oncogenic activities (red “minus” symbol). This is easier in the case of bifunctional genes whose products have concordant outputs, while it can be ineffective or even deleterious in the case of a discordant output.

In addition, we highlight that concordant or discordant molecular activities result in a concordant or discordant impact on tumorigenesis: the protein and the non-coding RNA can be both tumor suppressors or both oncogenes, but oncogenic and tumor-suppressive activities can also coexist (see Fig. 5 for specific examples).

How can we disentangle the specific role exerted by coding and non-coding products of the same genetic unit, when we face such a degree of molecular and biological complexity?

To ablate the genetic unit through classic homologous recombination-mediated knockout is a coarse approach that will most certainly lead to profoundly misleading results, due to the concomitant inactivation of two or more players. However, the knockout of the bifunctional gene can be coupled with the add-back of one of its functional products at the time. This strategy suffers from the limitation of triggering the expression of the added-back (non-)coding RNA at supra-physiological or under-physiological levels. Nevertheless, it helps define the function of each product per se, which should precede the study of functional cross-talks among multiple products.

Alternatively, more precise ways to remodel the genome can be used, such as CRISPR-mediated editing. For example, CRISPR technology allows to surgically mutagenize the MREs present in the 3′UTR of a given mRNA, preventing its ability to work as ceRNA. Nevertheless, pitfalls hide even behind these apparently “cleaner” genetic interventions. If the coding and the non-coding RNAs regulate each other’s expression, then the alteration of one will inevitably affect the other as well. Therefore, it will not be possible to establish whether the functional outcome is a direct or indirect consequence of the alteration introduced. Going back to the example mentioned above, the removal of MREs will certainly deprive the mRNA of its non-coding ceRNA activity *in trans*, but it will also have an effect *in cis*, as it will result in an increase in the stability/translation of the mRNA itself, and hence in the level of the corresponding protein, since it is no longer targeted by miRNAs.

Irrespectively of the approach used to study a bifunctional gene in vitro, we emphasize the importance of corroborating the obtained results with appropriate in vivo models. So far the in vivo characterization of non-canonical functions of coding and non-coding RNAs has been almost exclusively performed in xenograft models. Transgenic mouse models can be counted on one hand, while knockout mouse models are even fewer and all fall outside cancer research. For example, Masumoto et al.^210^ set up a knockout GEM to study the role played by *LINC00961* and its encoded polypeptide SPAR (Small regulatory Polypeptide of Amino acid Response) in muscle regeneration. Xenografted animals are easy to handle, and they produce quite consistent results in a relatively short time. Nevertheless, we should rely on them just as a first readout of cell autonomous outcomes. To fully grasp the involvement of the most promising (non-)coding candidates on disease/cancer initiation, progression, and response to pharmacological treatment, it is then necessary to develop appropriate GEMs in immunocompetent hosts.^91,211^

Moving from basic to translational research, bifunctional genes offer concrete therapeutic opportunities. This rests on the flourishing interest in RNA-based drugs, which are under investigation in alternative to or in combination with “classical” chemical inhibitors of protein function and have gained full acceptance during COVID-19 emergency.^212,213^ Bifunctional genes that produce only tumor suppressors or only oncogenes are easier to deal with. By contrast, bifunctional genes that produce one tumor suppressor and one oncogene need to be approached with caution: we must avoid the unintended impairment of the tumor-suppressive activity or the boosting of the oncogenic activity caused by our intervention.

In conclusion, we show that the study of protein-coding and non-coding products, their functions and regulation is essential to understand many genetic units in our genome. We also propose that bifunctional genes should be classified as such, distinguishing them from only coding ones and only non-coding ones. However, we point out that much more functional validation is needed towards such classification, both in vitro and in vivo, using the appropriate technological tools for genetic manipulation currently available or to be developed in the foreseeable future. We cannot deny that some intricacies are so thick that may prove daunting or almost impossible to resolve. Nonetheless, we are confident that the widespread transcription and partly overlapping translation of our genome is paving the way for unprecedented opportunities of discovery, drug development and disease treatment.
